# The impact of intimate partner violence on the trajectory of perinatal depression: a cohort study in a Chinese sample

**DOI:** 10.1017/S2045796020000463

**Published:** 2020-06-02

**Authors:** Fengsu Hou, Xingyu Zhang, Catherine Cerulli, Wenjun He, Yushi Mo, Wenjie Gong

**Affiliations:** 1 Department of Public Health, Shenzhen Kangning Hospital, Shenzhen, Guangdong Province, China; 2 Department of Psychiatry, University of Rochester Medical Center, Rochester, NY, USA; 3 Department of Systems, Populations and Leadership, School of Nursing, University of MichiganSchool of Nursing, University of Michigan, Ann Arbor, MI, USA; 4 Injury Control Research Center for Suicide Prevention, University of Rochester Medical Center, Rochester, NY, USA; 5 Susan B. Anthony Center, University of Rochester, Rochester, NY, USA; 6 Department of Medical Statistics and Epidemiology, School of Public Health, Sun Yat-sen University, Guangzhou, Guangdong Province, China; 7 Xiangya School of Public Health, Central South University, China

**Keywords:** EPDS, intimate partner violence, perinatal depression, pregnancy, prognosis

## Abstract

**Abstract**

**Aims:**

Intimate partner violence (IPV) is an important risk factor for perinatal depression (PND). But IPV's impact on the natural prognosis of PND symptoms is not well understood. We tested two hypotheses: (1) pregnant women with IPV experiences will exhibit more severe PND symptoms than women without IPV experience; (2) IPV experience will impede the recovery prognosis of PND. We also explored the contribution of IPV to PND comparing with other risk factors.

**Method:**

The sample is comprised of 813 pregnant women followed through perinatal period in Hunan, China. We assessed IPV experience using items from the Short Form of the Revised Conflict Tactics Scale (CTS2S), and PND symptoms via the Edinburgh Postnatal Depression Scale (EPSD). We conducted Linear Mixed-effects Model to compare the trajectories of PND symptoms between victims and non-victims and a multistage Generalised Estimating Equations Model to explore salient factors on the trajectory of PND symptoms.

**Results:**

There were 90 participants (11.07%) who reported IPV experience in the past 12 months. With respect to physical, psychological and sexual violence, the prevalence was 4.55% (37/813), 9.23% (75/813) and 2.34% (19/813). Victims reported more severe PND symptoms (*t* = 5.30, *p* < 0.01) and slower decreasing slope of trajectories (*t* = 28.89, *p* < 0.01). The PND trajectory was associated with IPV experience (OR = 3.78; 95% CI 1.39–10.26), social support (OR = 0.93; 95% CI 0.88–0.97), positive coping strategies (OR = 0.85; 95% CI 0.80–0.91), negative coping strategies (OR = 1.25; 95% CI 1.14–1.37) and monthly income of $0.15–$298.36 (compared to no income, OR = 0.0075; 95% CI 0.00052–0.11).

**Conclusions:**

The findings suggest the reported prevalence of IPV is lower in Hunan than most of the previous studies during perinatal period in other provinces of China, and IPV victimisation is associated with increased severity and slowed prognosis of PND symptoms. Future studies that screen for victimisation and establish its explicit mechanism to the poorer prognosis of PND symptoms would benefit the prevention and treatment of PND.

## Introduction

More than one-third (35%) of women worldwide have experienced physical and/or sexual violence at some time in their lives, with intimate partner violence (IPV) being the most common form of violence, which indicates perpetrators are current or former intimate partners (World Health Organization, 2012, 2013). IPV against women has been globally recognised as a severe challenge to women's health, safety and equity, with a lifetime prevalence ranging from 23.2 to 37.7% (García-Moreno *et al*., 2013). Specifically, the prevalence of IPV during the perinatal period ranges from 1 to 35% worldwide, and can result in several significant adverse physical, psychiatric and neurological health problems for women and newborns (Gazmararian *et al*., 2000; World Health Organization, 2002, 2013; García-Moreno *et al*., 2005; Devries *et al*., 2010; Shah and Shah, 2010; Alhusen *et al*., 2015; Halim *et al*., 2017). Due to cultural barriers, scholars have overlooked IPV in China for decades. There are few studies reporting the prevalence of IPV against women during the perinatal period, with no national data available. The most recent meta-analysis included all 13 published studies in this area among Chinese population, and reported the overall prevalence of IPV during pregnancy was 7.7%; furthermore, none of these studies investigated the impact of IPV on the trajectory and the prognosis of perinatal depression (PND) (Wang *et al*., 2017).

Among the health outcomes of IPV during the perinatal period, PND is one of the most common long-term chronic health outcomes and a growing public health concern. This is due to two reasons. Depression is the sixth largest contributor to the global burden of disease among females aged from 15 to 45 years old (Institute for Health Metrics and Evaluation, 2018). Also, PND is often underdiagnosed and can result in a series of adverse outcomes for women, children and families (Campbell, 2002; Bansil *et al*., 2010; Alhusen *et al*., 2015; Allbaugh *et al*., 2015; Halim *et al*., 2017). The prevalence of PND varies from 7 to 25% during the antepartum period and 10 to 20% during the postpartum period (Leung and Kaplan, 2009; Gelaye *et al*., 2016). Comparing with the general female population, the prevalence of PND was higher among IPV victims ranging from 15 to 79.2% (Leung *et al*., 2002; Cerulli *et al*., 2011; Islam *et al*., 2017). Many studies that have been conducted are cross-sectional and make it difficult to assess the causal relationships between IPV and PND. Further, both have similar adverse health outcomes, including miscarriage, preterm delivery, antepartum haemorrhage and low birth weight (Bansil *et al*., 2010; Beydoun *et al*., 2010; Alhusen *et al*., 2015; Doyle *et al*., 2015; Jackson *et al*., 2015; Finnbogadottir *et al*., 2016; Halim *et al*., 2017).

Recent studies among PND patients have revealed that the trajectory of symptoms will alleviate over time (Ahmed *et al*., 2018; Drozd *et al*., 2018). Of note, the trajectory of PND refers to the longitudinal pattern of PND. However, it is not clear whether IPV victimisation will interfere with the natural prognosis of PND, which might be overlooked during treatments. Hence, we designed this study to test two hypotheses: (1) pregnant women who have experienced IPV will exhibit more severe PND symptoms than women without any IPV experiences; (2) IPV experience will impede the recovery prognosis of PND. We also explored the contribution of IPV to PND comparing with other risk factors.

## Methods

Data for this study come from a larger cohort study intended to develop a prediction model for PND in Hunan Province, central south of China.

### Sample and sampling

From September 2016 to February 2017, the parent study recruited pregnant women by convenient sampling from two maternal and child health care hospitals in Changsha City, the capital city of the province, and Yiyang City, a less economically developed area of Hunan (Hunan Bureau of Statistics, 2018). In the recruiting period, interviewers invited all pregnant women, who showed up at the obstetrical clinic of each hospital, to participate in the study. Once provided oral consent, interviewers would further screen for the eligibility, and the inclusion criteria were: (1) being 18 years old and above; (2) being within 13 weeks of gestation; (3) planning to take antenatal and postpartum examinations and care in the two hospitals; and 4) providing written consent and agreeing to participate in the follow-up interviews. All participants participated voluntarily without receiving any financial or treatment incentives. We excluded women who showed severe pregnancy complications or any severe mental/cognitive problems that would impede them finishing the survey, including schizophrenia, paranoia, autism, dementia and mental retardation. We also excluded participants who did not provide information on their IPV experiences for this analysis.

### Procedure

According to the Code of National Basic Public Health Service of China, maternal and child health care hospitals are required to provide perinatal examinations for pregnant women seven times: any time before the gestational week 12, during the gestational week 16–20, 21–24, 28–36 and 37 to 40, any time in the postpartum week 1 and week 6 (National Health and Family Planning Commission of People's Republic of China, 2017). Therefore, the parent study used seven time points for data collection – T1 (baseline): when participants came to the hospitals for their first antenatal examinations (gestational week 4–12), T2 (gestational week 17–20), T3 (gestational week 21–24), T4 (gestational week 31–32), T5 (gestational week 35 till delivery), T6 (day 7 after delivery) and T7 (week 6 after delivery).

Once the participants provided written consent, experienced and well-trained nurses would help participants complete the survey. When participants finished the survey, nurses would check if all required fields had been completed and remind participants to complete the missing items before participants left unless they refused.

### Assessments

As the surveys were completed after medical examinations coupled with the length of questionnaires, the parent study collected different information, except PND, which was measured seven times, at different time points to save participants' time and reduce stress. The content of assessments at each time point is showed in Table 1.

**Table 1.** The content of assessments at each time point

Assessments	T1	T2	T3	T4	T5	T6	T7
Sociodemographic information	√						
The Short Form of the Revised Conflict Tactics Scale (CTS2S)			√				
The Edinburgh Postnatal Depression Scale (EPDS)	√	√	√	√	√	√	√
The Brief Resilience Scale (BRS)			√				
The Simplified Coping Style Questionnaire (SCSQ)				√			
The Social Support Rating Scale (SSRS)					√		

The self-reported questionnaires collected sociodemographic information including age, education, monthly income, family structure, first pregnancy, abortion history, smoking, drinking, the satisfaction of current relationship, and partners' age, education and monthly income at the T1.

We adopted three items from the victimisation subscale of the Short Form of the Revised Conflict Tactics Scale (CTS2S, Chinese version, 2014) to investigate participants' IPV experience at T3, which shows good reliability and validity (Straus and Douglas, 2004; Zhang *et al*., 2014). Considering IPV is still seen as private conflicts which should be discussed within the family, and it may increase tension between participants and research staff, we first embedded the items in the Brief Resilience Scale (BRS), and then added positive relationship statements to increase comfort during the survey, for example: ‘He accompanies me to do the gestational examination and shows great attention to my test results’. We used one item for physical violence (‘my partner punched or kicked or beat-me-up’), one item for psychological violence (‘my partner insulted or swore or shouted or yelled at me’) and one item for sexual violence (‘my partner used force to make me have sex, or my partner insisted on sex when I did not want to or insisted on sex without a condom’). Considering the CTS2S does not investigate a common form of psychological violence in China, known as the ‘cold violence’ or the cold-shoulder from partners (Wu *et al*., 2005), we used the statement ‘my partner used to ignore me or pay no attention to me for a long time’. Participants who endorsed any of the four items were considered to be IPV victims.

We used the Mandarin version of the Edinburgh Postnatal Depression Scale (EPDS), which has shown great reliability and validity in screening for depressive symptoms in the perinatal period, to explore participants' PND symptoms at each study time point (Cox *et al*., 1987; Murray and Cox, 1990; Evins and Theofrastous, 1997; Lee *et al*., 1998; Gibson *et al*., 2009; Lau *et al*., 2010; Shrestha *et al*., 2016). The total score ranges from 0 to 30, and a higher score indicates a higher severity of PND symptoms. The Cronbach's *α*s were 0.77, 0.81, 0.81, 0.82, 0.83, 0.84 and 0.85, respectively.

We used the BRS to explore participants' ability to recover from stress at T3, which consists of three positive statements and three negative statements (Smith *et al*., 2008). The total score ranges from 6 to 30, and a higher score indicates a better ability to recover from stress. The Cronbach's *α* was 0.75.

We employed the Simplified Coping Style Questionnaire (SCSQ) to explore participants' coping strategies for dealing with daily stress at T4, developed in China, measuring positive and negative coping (Xie, 1998). The mean score of each subscale indicates the tendency to use the coping strategy, a higher score indicating a higher tendency. The Cronbach's *α* was 0.84.

Participants answered the Social Support Rating Scale (SSRS) to explore their social support at T5, which was developed and widely used in China with great reliability and validity and was consisted of objective social support, subjective social support and the utilisation of social support subscales (Xiao, 1993; Liu *et al*., 2008; Su *et al*., 2009; Xie *et al*., 2009). The total score ranges from 12 to 66 with a higher score indicating a higher level of social support. The Cronbach's *α* was 0.88.

### Statistical analysis

In this study, we analysed data with R (version 3.5.1) and we set the statistical significance at 0.05 (R Core Team, 2013).

#### Primary analysis

The primary analysis compared the trajectory of PND symptoms between participants with and without IPV victimisation experience. Because the perinatal examination services were not mandatory, pregnant women might not attend examinations during the perinatal period or visit other service providers. As a result, there was some missing data. We conducted a pilot analysis to compare sociodemographic characteristics between participants with and without missing data in EPDS, and the results indicated there were no sociodemographic differences, including IPV experience, between the two groups of participants. Hence, if a participant's EPDS score was missing in the follow-ups, we would complete it with the LOCF (last observation carried forward) method (Overall *et al*., 2009).

First, we conducted one-way ANOVA to compare the EPDS scores at each time point between IPV victims and non-victims; we also conducted mixed-model ANOVA to compare the differences between the two groups as a repeated measurement. To compare the trajectories between IPV victims and non-victims, we conducted Linear Mixed-effects Model analysis. We applied the R package ‘psych’, ‘lm4’ and ‘lmerTest’ to perform analysis (Bates *et al*., 2015; Kuznetsova *et al*., 2017; Revelle, 2017).

#### Secondary analysis

The secondary analysis explored the contribution of IPV to PND controlling for other associated factors. The dependent variable was repeated measured EPDS score; the independent variables were IPV experience, resilience, coping skills and social support. If there were any missing values of the BRS, the SCSQ and the SSRS, we would impute the missing score with the mean score of each scale.

The covariates were participants' and their partners' ages and education levels (junior high and below, high school, Bachelor's degree and master's degree and above). We also included both parties' monthly income (no income, $0.15–$298.36, $298.51–$745.90, $746.05–$1491.80 and ⩾$1491.95), family structure (nuclear family, stem family with parents-in-law, stem family with parents, living alone and others), first pregnancy, abortion history, smoking and drinking history, and the satisfaction of current relationship (satisfied, OK and not satisfied).

We used the Generalised Estimating Equations (GEE) Model to explore the contributions of factors to the trajectory of PND by R package ‘geepack’ (Halekoh *et al*., 2006). We conducted univariate GEE analysis first, and then conducted multivariate GEE analysis with the factors of significance in the univariate analysis. We chose the Quasi-likelihood under Independence Model Criterion (QIC) as the goodness of fit to assess models, and a smaller value of QIC indicated a better model (Cui, 2007). We calculated the generalised variance inflation factor (GVIF), the GVIF^(1/2*Df) value of 2 and above indicating the multicollinearity problem (Fox *et al*., 2007). Odds ratios (ORs) and 95% CIs were estimated to determine the associations between factors and the trajectory of PND symptoms.

#### Descriptive analysis

To compare the characteristics of IPV victims and non-victims, we conducted one-way ANOVA for continuous variables (age, social support, resilience and coping skills), *χ*^2^ test for categorical variables (education, income, first pregnancy, abortion history, smoking, drinking, satisfaction of current relationship and family structure) and Fisher's exact test if needed. Descriptive analysis was conducted by R package ‘psych’ (Revelle, 2017).

### Ethics

The protocol including the written consent was reviewed and approved by the Ethics Committee of Central South University (No.CTXY-140002-2). During analysis, we utilised de-identified data.

In this study, nurses would immediately calculate the EPDS score and check for IPV experience after the survey at each time point. At any time, if participants received a total score ⩾9 indicating a high risk for depression, we would explain the result and help them transfer to a local psychiatrist. Meanwhile, if participants showed any IPV experience, we would provide and explain printed materials, including a list of helpful resources and contact information, manuals of utilizing these resources and an introduction of IPV.

## Results

The parent study recruited 1126 participants, with 813 participants (72.2%) eligible for this study. Participants aged from 20 to 46 years, with a mean of 30.78 ± 4.39 years. A total of 550 participants (67.65%) had received bachelor's degree and above; 395 participants (48.59%) had monthly income ranged from $298.51 to $745.90; 576 participants (70.85%) claimed this was not their first pregnancy; and among the 578 participants who had provided abortion history information, 407 of them (70.42%) had an abortion before. There were 31 (3.81%) and 66 (8.12%) participants who reported smoking and drinking, respectively. The score of BRS ranged from 6 to 29, with a mean of 18.59 ± 2.12. In the SCSQ, the score of positive coping ranged from 0 to 36, with a mean of 24.30 ± 5.56; and the score of negative coping ranged from 0 to 24, with a mean of 9.79 ± 3.86. The score of SSRS ranged from 13 to 60 with a mean of 43.50 ± 6.20. There were 660 participants (81.18%) that reported satisfaction with their current relationship and 326 participants (40.10%) were living in a nuclear family. The age of participants' partners ranged from 18 to 48 years, with a mean of 30.78 ± 5.01 years. Among participants' partners, 522 of them (64.12%) had received a bachelor's degree and above; 352 of them (43.42%) had monthly income ranged from $298.51 to $745.90. See Table 2 for details.

**Table 2.** Demographic characteristics of 813 participants

Characteristics	Total *N* = 813	Victims *n* = 90	Non-victims *n* = 723	*p*
Age (mean, s.d.)	30.78 (4.39)	31.21 (4.51)	30.73 (4.37)	<0.01
Education				0.48
Junior high and below	86	12	74	
High school	176	22	154	
Bachelor's degree	477	46	430	
Master's degree and above	74	10	64	
Personal monthly income*,^#^				0.87
No income	217	22	195	
$0.15–$298.36	41	6	35	
$298.51–$745.90	395	46	349	
$746.05–$1491.80	118	12	106	
⩾$1491.95	24	3	21	
First pregnancy*				0.003
No	576	76	500	
Yes	219	12	207	
Abortion*				1
No	171	22	149	
Yes	407	52	355	
Smoking*,^#^				0.24
No	776	88	688	
Yes	31	1	30	
Drinking*				0.75
No	746	80	666	
Yes	66	8	54	
Resilience (mean, s.d.)	18.59(2.12)	18.32 (2.42)	18.62 (2.08)	<0.01
Coping skills* (mean, s.d.)				
Positive coping	24.31 (5.56)	22.82 (5.98)	24.49 (5.48)	<0.01
Negative coping	9.79 (3.86)	10.72 (4.49)	9.68 (3.76)	0.016
Social support*	43.50 (6.20)	39.74 (8.10)	43.93 (6.74)	<0.01
Satisfaction of current relationship*,^#^				<0.01
Satisfied	660	58	602	
OK	140	26	114	
Unsatisfied	3	2	1	
Family structure*,^#^				0.63
Nuclear family (the couple, with or without offsprings)	326	37	289	
Stem family with parents-in-law	276	28	248	
Stem family with own parents	112	11	101	
Living along	73	7	66	
Others	19	4	15	
Partner's age*	30.78 (5.01)	31.68 (5.12)	30.67 (4.99)	<0.01
Partner's education*,^#^				0.90
Junior high and below	92	8	84	
High school	188	19	169	
Bachelor's degree	451	51	400	
Master's degree and above	71	8	63	
Partner's monthly income*,^#^				0.31
No income	16	3	13	
$0.15–$298.36	21	0	21	
$298.51–$745.90	352	35	317	
$746.05–$1491.80	308	37	271	
⩾$1491.95	104	12	92	

Note: ‘*’ indicates missing values; ‘#’ indicates Fisher exact test.

### Intimate partner violence experience

Overall, 90 participants reported IPV experience in the past 12 months, with a prevalence of 11.07% (90/813). For physical, psychological and sexual violence, the prevalence was 4.55% (37/813), 9.23% (75/813) and 2.34% (19/813), respectively.

Comparing with non-victims, we found victims were older (32.21 ± 4.39 *v*. 30.73 ± 4.37 years) and their partners were also older (31.68 ± 5.12 *v*. 30.67 ± 4.99 years). The prevalence of first-time pregnancy was lower in victims (13.64 *v*. 29.28%). Victims had lower scores in the BRS (18.32 ± 2.42 *v*. 18.62 ± 2.08) and in the positive sub-scale of the SCSQ (22.82 ± 5.98 *v*. 24.49 ± 5.48). They also had higher scores in the negative sub-scale of the SCSQ (10.72 ± 4.49 *v*. 9.68 ± 3.76), indicating less resilience to stress and were more likely to adopt negative strategies. Victims had lower social support levels than non-victims (39.74 ± 8.10 *v*. 43.93 ± 6.74). See Table 2 for details.

### Perinatal depression symptoms and the trajectories

Among all participants, the mean score of EPDS decreased from T1 to T6, and then slightly increased at T7. The mean scores of EPDS were 8.60, 8.22, 7.99, 7.90, 7.87, 7.26 and 7.60, respectively. The mixed-model ANOVA analysis did show the victims had higher scores on the EPDS than non-victims at all time points (*F* = 28.11, *p* < 0.01), indicating a higher level of depression symptoms through the whole perinatal period. See Table 3.

**Table 3.** The comparison of EPDS scores between victims and non-victims during follow-ups

Time point	Total (mean, s.d.)	Victims (mean, s.d.)	Non-victims (mean, s.d.)	*F*^a^	*p*
T1	8.60 (4.00)	10.00 (3.68)	8.43 (4.00)	12.50	<0.01
T2	8.22 (4.37)	10.34 (5.09)	7.95 (4.19)	26.29	<0.01
T3	7.99 (4.20)	10.66 (4.81)	7.66 (4.00)	42.75	<0.01
T4	7.90 (4.22)	10.04 (4.85)	7.63 (4.06)	26.98	<0.01
T5	7.87 (4.27)	9.54 (4.72)	7.66 (4.16)	15.90	<0.01
T6	7.26 (4.58)	8.82 (4.62)	7.07 (4.54)	11.89	<0.01
T7	7.60 (4.72)	9.21 (4.53)	7.41 (4.70)	11.86	<0.01
*F*^b^	28.11				
p	<0.01				

a One-way ANOVA test at each time point between victims and non-victims.

b Mixed-model ANOVA test for repeated measurements between victims and non-victims.

For victims, the mean score of EPDS increased from 10.00 at T1 to 10.66 at T3, then decreased to 8.82 at T6, and eventually increased to 9.21 at T7. For non-victims, the mean score of EPDS showed a similar trajectory over time points that decreased from 8.43 at T1 to 7.07 at T6 and slightly increased to 7.41 at T7.

**Fig. 1.**
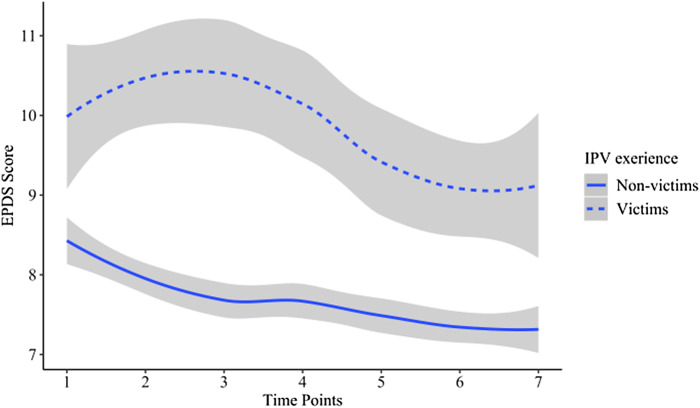
The trajectories of perinatal depression between IPV victims and non-victims.

We compared the trajectories of PND between victims and non-victim, and the result showed that the trajectories were significantly different between the two groups of pregnant women (*t* = 5.30, *p* < 0.01). The decreasing slope for victims' PND trajectory was slower than non-victims' (*t* = 28.89, *p* < 0.01) (Fig. 1).

### The trajectory of PND and associated factors

In univariate analysis, participants' age, education, social support, coping skills, their partners' age and monthly income, and IPV experience were associated with the trajectory of PND with *p* < 0.05 (Table 4). We included these factors in the multivariate analysis (Model 1). Results showed that participants' age and education, and their partners' age were not significant with a QIC of 5443. Hence, we excluded these covariates in Model 2, and the results showed the remaining factors were all significantly associated with PND and the QIC was 5423 (Table 5). The values of GVIF^(1/2*Df) for IPV experience, social support, positive and negative coping skill, and partner's age and monthly income were 1.03, 1.01, 1.04, 1.16, 1.17 and 1.06, respectively, indicating a good model fitness. We found that if social support and the incline of using positive coping strategies increased 1 unit, pregnant women would have 0.93 (OR = 0.93; 95% CI 0.88–0.97) and 0.85 times (OR = 0.85; 95% CI 0.80–0.91) the risk to have PND symptoms, respectively. Comparing with pregnant women whose partners had no monthly income, when their partners had monthly income ($0.15–$298.36), they had 0.0075 times the risk to have PND symptoms (OR = 0.0075; 95% CI 0.00052–0.11). Contrarily, if the incline of using negative coping strategies increased 1 unit, pregnant women would have about 1.25 (OR = 1.25; 95% CI 1.14–1.37) times the risk to have PND symptoms; and if having any IPV experiences, pregnant women would have 3.78 times (OR = 3.78; 95% CI 1.39–10.26) the risk to have PND symptoms.

**Table 4.** The univariate GEE analysis of perinatal depression and associated factors

Factors	OR	Wald	95%CI	
			Lower	Upper
Age	0.90	6.06	0.83	0.98
Education				
Junior high and below	–	–	–	–
High school	0.36	1.71	0.076	1.68
Bachelor's degree	0.39	1.55	0.088	1.72
Master's degree and above	0.13	4.72	0.020	0.82
Personal monthly income				
No income	–	–	–	–
$0.15–$298.36	0.38	1.62	0.088	1.685
$298.51–$745.90	0.46	2.52	0.18	1.20
$746.05–$1491.80	0.48	1.06	0.12	1.92
⩾$1491.95	0.39	1.83	0.10	1.52
First pregnancy				
No	–	–	–	–
Yes	4.54	0.66	0.12	174.78
Abortion				
No	–	–	–	–
Yes	0.81	0.23	0.34	1.94
Smoking	2.76	0.44	0.14	55.96
Drinking	2.59	1.40	0.53	12.53
Social support	0.85	33.30	0.81	0.90
Resilience	1.08	0.35	0.83	1.42
Coping skills				
Positive coping	0.85	23.90	0.80	0.91
Negative coping	1.17	8.4	1.05	1.30
Satisfaction of current relationship				
Satisfied	–	–	–	–
OK	0.94	0.13	0.67	1.33
Not satisfied	0.32	0.70	0.082	1.28
Living condition				
Nuclear family	–	–	–	–
Nuclear family with parents-in-law	1.42	0.60	0.58	3.49
Nuclear family with own parents	1.54	0.58	0.51	4.71
Living along	1.08	0.01	0.26	4.49
Others	0.74	0.07	0.084	6.54
Partner's age	0.91	8.39	0.90	0.91
Partner's education				
Junior high and below	–	–	–	–
High school	0.53	1.00	0.15	1.84
Bachelor's degree	0.97	0.00	0.33	2.81
Master's degree and above	0.31	2.53	0.071	1.32
Partner's monthly income				
No income	–	–	–	–
$0.15–$298.36	0.0023	7.80	0.000033	0.16
$298.51–$745.90	0.07	1.60	0.0012	4.29
$746.05–$1491.80	0.11	1.14	0.0018	6.34
⩾$1491.95	0.03	2.97	0.00042	1.66
IPV experience				
No	–	–	–	–
Yes	7.06	10.9	2.21	22.58

**Table 5.** The multivariate GEE analysis of perinatal depression and associated factors

Factors	Model 1				Model 2			
	OR	Wald	95% CI		OR	Wald	95% CI	
			Lower	Upper			Lower	Upper
Age	0.96	0.55	0.85	1.09	–	–	–	–
Education								
Junior high and below	–	–	–	–	–	–	–	–
High school	0.64	0.41	0.16	2.55	–	–	–	–
Bachelor's degree	1.04	0.00	0.25	4.23	–	–	–	–
Master's degree and above	0.98	0.44	0.16	5.81	–	–	–	–
Social support	0.92	9.35	0.88	0.97	0.93	8.75	0.88	0.97
Coping skills								
Positive coping	0.85	26.61	0.81	0.91	0.85	25.93	0.80	0.91
Negative coping	1.25	22.89	1.14	1.37	1.25	22.67	1.14	1.37
Partner's age	0.98	0.11	0.89	1.09	–	–	–	–
Partner's monthly income								
No income	–	–	–	–	–	–	–	–
$0.15–$298.36	0.0082	12.99	0.00060	0.11	0.0075	12.91	0.00052	0.11
$298.51–$745.90	0.12	2.71	0.011	1.49	0.12	2.61	0.0095	1.57
$746.05–$1491.80	0.21	1.37	0.017	2.81	0.22	1.32	0.017	2.92
⩾$1491.95	0.057	4.52	0.0040	0.80	0.06	4.21	0.0041	0.88
IPV experience								
No	–	–	–	–	–	–	–	–
Yes	3.78	6.92	1.40	10.26	3.78	6.67	1.39	10.26
QIC	5443				5423			

## Discussion

To our knowledge, this is the first study in China to recruit a cohort of pregnant women to explore the impact of IPV on the trajectory and prognosis of PND symptoms. In this study, we reported the prevalence of IPV was 11.07% in the past 12 months, and the prevalence of physical, psychological and sexual violence was 4.55, 9.23 and 2.34%, respectively. We also reported that IPV experience was positively associated with the severity of PND symptoms; victims exhibited more severe PND symptoms and recovered more slowly from PND symptoms than non-victims.

The reported overall prevalence of IPV was lower than most of the previous studies during the perinatal period which ranged from 16.8 to 22.6% in Tianjing, Liaoning, Henan and Shannxi Province, but it was higher than the reported prevalence of 6.4% in Guangdong (Fan *et al*., 2006; Wu *et al*., 2006). Currently, IPV during perinatal period is not well understood in China, given the limited number of studies and a wide range of reported prevalence. The prevalence rate variation can be explained by regional cultural beliefs that lead to different understandings of IPV, inaccurate reporting, non-unified surveys that vary in sensitivity and reliability, and limited samples. Meanwhile, due to the lack of a standardised treatment protocol for IPV among health providers in China, IPV remains a very sensitive topic for both health providers and potential victims. There is a mutual hesitancy to discuss or disclose IPV. Hence, caution is warranted in interpreting these findings.

We believed the elevated EPDS scores among victims illustrated the psychological impact of IPV (Campbell and Lewandowski, 1997; Campbell, 2002; Devries *et al*., 2013), and indicated the traumatic experience was a risk factor for PND symptoms. Consistent with previous studies, we found victims were nearly four times likely to have more severe PND symptoms (Bonomi *et al*., 2009; Ludermir *et al*., 2010; World Health Organization, 2011; Melo *et al*., 2012). In this study, the trajectory of PND symptoms decreased from being pregnant to delivery and then increase after delivery regardless of IPV experience, which was consistent with trajectory analyses of PND (Mora *et al*., 2008; Christensen *et al*., 2011; Ahmed *et al*., 2018). Meanwhile, victims showed more severe depressive symptoms and a slower prognosis of PND symptoms. Because the prognosis could only be observed through multi-metering at different times, evidence from cross-sectional studies would overlook the impact on the perinatal period; before, during and after the birth. Thus, this study provides valuable insights for clinicians that they should consider not only the possible bidirectional causal associations between IPV experience and PND but also the impact of IPV on the duration and outcomes of PND symptoms when victims’ symptoms recover more slowly than non-victims.

We noticed an increase of PND symptoms from T1 to T3 followed by a decline till T6 among victims. To explain this peak at T3 when we investigated IPV experience, we reviewed the survey procedure and confirmed participants completed the EPDS before IPV items, which excluded distress caused by the re-experience of victimisation. Combining these findings, we suspect an IPV experience would worsen pregnant women's PND symptoms. This suggests it is important to screen women with PND for victimisation during perinatal examinations, especially at T3, and then to provide interventions targeting IPV. This, in turn, may improve victims' mental health to the utmost.

We found a higher level of social support was associated with less severity of PND symptoms. According to the Buffer Theory, social support plays a protective role in everyday life that buffers the stress or impact of adverse events (Cassel, 1976; Cochrane and Stopes-Roe, 1981; Schaefer *et al*., 1981). Our previous studies reported the perceived/subjective social support played a more important role in preventing depression over other dimensions of social support; and, overall, social support protected Chinese women from IPV, which could indirectly protect women from PND (Hou *et al*., 2015, 2018).

We believe that a specific cultural tradition that starts right after delivery and lasts for 1 month, known as ‘*zuo yue zi*’ or ‘doing the month’, increases women's social support. The tradition requires new mothers to be confined for 1 month for recovery. The new mother becomes the focus of the whole family and must avoid showering, catching a cold, eating cold food, doing housework (even taking care of the newborns except breast feeding), outdoor activities and should strictly stay in bed and take as much rest as possible. Women also follow the traditional Chinese medicine diet instructions (Holroyd *et al*., 1997; Raven *et al*., 2007). Hence, during this tradition, women could receive relief, satisfaction and joy that buffer depressive symptoms; meanwhile, the family's help in daily affairs and being the focal point would increase perceived and instrumental social support, which also buffers depressive symptoms. This may explain the lowest EPDS score during the first postpartum week. However, this tradition also isolates women from daily social interactions and reduces their activities, which could lead to the deterioration of social network and physical functions at the late stage of ‘*zuo yue zi*’. When this tradition ends, new mothers would take care of the newborns and prepare to return to work, which may lead to the increased depressive symptoms at postpartum week 6. We did not explore the relationship between this tradition, IPV and PND in the current study. The relationship between ‘zuo yue zi’ and IPV has not been reported anywhere yet, and it is a common Chinese cultural tradition. We plan to explore our hypothesis of ‘zuo yue zi’ and PND in the future, and we encourage other researchers to explore this as well.

We found victims were inclined to use negative coping skills that were positively associated with PND symptoms. Women with PND were inclined to use non-adaptive/negative coping strategies to deal with stress during perinatal period, including avoidance, distancing, denial, self-blame, substance use and behavioural disengagement. These strategies were associated with the health outcomes of IPV experience, especially PTSD, depression, substance use and re-victimisation in return (Kocot and Goodman, 2003; Faisal-Cury *et al*., 2004; Pakenham *et al*., 2007; Iverson *et al*., 2013; Flanagan *et al*., 2014; Gutiérrez-Zotes *et al*., 2016; Azale *et al*., 2018). Thus, we believe coping skills would not only interfere with IPV experience and its health outcomes, but also with stress and depressive symptoms.

Pregnant women with partners who had no income in this study were more likely to develop higher levels of PND symptoms, similar to studies in the general population that showed lower household income was positively associated with depression (Kahn *et al*., 2000; Lorant *et al*., 2007; Pan *et al*., 2008). We interpret a partner with no income as unemployed or doing housework, and under this circumstance, pregnant women would perceive severe financial stress for household finances during the perinatal period and in the long term. Of note, the imbalance of income between couples and poverty was a risk factor of IPV (Jewkes, 2002; World Health Organization, 2010); thus, financial stress could indirectly lead to PND. Further, there were 16 pregnant women with partners who had no income, and 14 of them did not have income either. The 14 participants were younger (28.43 ± 3.82 *v*. 30.83 ± 4.38), less educated and had lower social support (38.86 ± 7.50 *v*. 43.57 ± 6.17) than the rest of the participants. Unfortunately, we believed the sample size of couples that had no income was too small to explore the impact on IPV and PND. We encourage future research to focus on this marginalised group of pregnant women regarding their mental health, IPV experiences as well as the relationship dynamics within the couple and its impact on both physical and mental health.

### Limitations

We recognise that there are a few study limitations worth noting. First, during recruiting, the study did not record how many pregnant women we have approached and how many of them refused to participate in the screen for eligibility. Hence it was difficult to estimate the representativeness of the sample compared with the population that the two hospitals served. Second, over two-thirds of the participants and their partners received a bachelor's degree or higher, which were higher than the average education level in China. Higher education level is associated with a lower risk for IPV experience that, in return, may have an impact on the results. Third, the parent study designed the follow-up time length from pregnancy until 42 days postpartum. However, we could not observe IPV's impact on the peak time of postpartum depression which was 2–3 months after delivery (Stuart-Parrigon and Stuart, 2014). Fourth, because we collected IPV information at T3 (gestational week 21–24), we could not differentiate the impact of IPV that happened between T1 and T3 from that happened before pregnancy on the trajectory of PND. We could overlook both new and reoccurring victimisations from T3 to the postpartum period and underestimate the impact of IPV on PND symptoms. Thus, the results must be interpreted carefully. At last, we understand that participants can interpret follow-up surveys on depressive symptoms as caring about their feelings, which, in return, could serve as an intervention for PND, which might have an impact on the trajectories of PND for all participants.

Thus, we encourage future longitudinal research to investigate the latent trajectories of PND among women who experience IPV before or during the perinatal period, to explore the relationship between victimisation and the peak of PND symptoms, and to understand the mechanism between IPV and PND eventually. Meanwhile, developing and applying a standardised universal screening tool for IPV through the whole perinatal period would benefit understanding the status in quo of IPV among pregnant women in China, which could lay the foundation for developing and implementing effective interventions.

## Conclusion

The findings suggest the reported prevalence of IPV is lower in Hunan than most of the previous studies during perinatal period in other provinces of China, and IPV victimisation is associated with increased severity and slowed prognosis of PND symptoms. Future studies that screen for victimisation and establish its explicit mechanism to the poorer prognosis of PND symptoms would benefit the prevention and treatment of PND.

## Data

The data that this manuscript is based are not available to the public. The data from this study are under certain restrictions according to the National Natural Science Foundation Committee of China and always under the supervision of the principal investigator of the study. Thus, there are access restrictions to the data. However, at any time, researchers can contact the principal investigator (Wenjie Gong, gongwenjie@csu.edu.cn) for data sharing.
